# Supplementary material for: Deciphering the explanatory potential of blood pressure variables on post-operative length of stay through hierarchical clustering: A retrospective monocentric study
Source: PLoS One. 2024 Sep 13;19(9):e0308910. doi: 10.1371/journal.pone.0308910 (PMC11398650; doi:10.1371/journal.pone.0308910)
Supplement: S1 Table — (DOCX) [file pone.0308910.s001.docx]

**S1_Table:** Construction of variables from arterial pressure. X being mean arterial pressure, diastolic arterial pressure, systolic arterial pressure, or pulse pressure

| Features | Formula | Description |
| --- | --- | --- |
| Min X | Min(X) | Minimal value reached during the intervention |
| Max X | Max(X) | Maximal value reached during the intervention |
| Drop X | Max(X)- Min(X) | Difference between the maximal and the minimal value computed over the entire intervention |
| Mean X | Mean(X) | Mean computed during the intervention |
| Median X | Median(X) | Median computed during the intervention |
| Std X | Std(X) | Standard deviation over the entire period |
| Var X | Std(X)/ Mean(X) | Variability of the signal |
| Cum time X>X0 | $\int{\mathbf{1}(t)}_{X<X0}\mathrm{dt}$  ${\mathbf{1}(t)}_{A}:indicator function$ | Cumulative time X stays below X0. For each variable, X0 are chosen in a range going from the 5^th^ to the 80^th^ percentile, iterating every 2 pts:  MAP[5 ;75]%= [61; 93]mmHg  DAP= [49; 71] mmHg  SAP= [82; 126] mmHg  PP= [30; 56 ] mmHg |
| Area time X>X0 | $-\int{\mathbf{(}X\left( t \right)-X0\mathbf{)1}(t)}_{X<X0}\mathrm{dt}$ | Cumulative area X stays below X0. For each variable, X0 are chosen in a range going from the 5^th^ to the 80^th^ percentile, iterating every 2 pts. |

MAP: mean arterial pressure; PP: pulse pressure; SAP: systolic arterial pressure; DAP: diastolic arterial pressure.

Min: minimal value observed during anesthesia; Max: maximal value observed during anesthesia; Drop: difference between max and min taken over anesthesia: Mean: mean of all values measured during anesthesia; Median: median of all values measured during anesthesia; Std: standard deviation of all values measured during anesthesia; Var: variability of all values measured during anesthesia; CumTime: cumulative time; AreaTime: cumulative area measurements*.*
