# Supplementary material for: Deciphering the explanatory potential of blood pressure variables on post-operative length of stay through hierarchical clustering: A retrospective monocentric study
Source: PLoS One. 2024 Sep 13;19(9):e0308910. doi: 10.1371/journal.pone.0308910 (PMC11398650; doi:10.1371/journal.pone.0308910)
Supplement: S2 Table — (DOCX) [file pone.0308910.s002.docx]

**S2_Table:** Absolute standardized mean distance per cluster

- Absolute standardized mean distance per cluster for Cluster 1

- Absolute standardized mean distance per cluster for Cluster 2

- Absolute standardized mean distance per cluster for Cluster 3

- Absolute standardized mean distance per cluster for Cluster 4

**Absolute standardized mean distance per cluster for Cluster 1**

| Cluster 1 |  |  |  |  |  |
| --- | --- | --- | --- | --- | --- |
| Variables | **Overall (9,516)** | **No pLOS (6,289)** | **pLOS (3,227)** | **p-val** | **aSMD[95%CI]** |
| Drop PP | 37.0 [25.7; 50.1] | 34.5 [24.3; 47.6] | 41.9 [29.4; 54.1] | <0.001 | 0.355 [0.312; 0.399] |
| Std PP | 8.0 [5.6; 11.2] | 7.4 [5.3; 10.4] | 9.0 [6.3; 12.2] | <0.001 | 0.333 [0.290; 0.377] |
| Drop SAP | 56.1 [43.0; 69.1] | 53.8 [40.9; 67.2] | 60.2 [47.9; 72.1] | <0.001 | 0.312 [0.271; 0.356] |
| Var PP | 0.2 [0.1; 0.2] | 0.2 [0.1; 0.2] | 0.2 [0.1; 0.2] | <0.001 | 0.312 [0.270; 0.355] |
| Max PP | 70.3 [59.5; 83.3] | 68.4 [58.3; 81.1] | 74.0 [62.0; 86.6] | <0.001 | 0.277 [0.231; 0.320] |
| Std SAP | 13.1 [10.0; 16.7] | 12.6 [9.7; 16.2] | 14.1 [10.9; 17.5] | <0.001 | 0.260 [0.220; 0.303] |
| Var SAP | 0.1 [0.1; 0.1] | 0.1 [0.1; 0.1] | 0.1 [0.1; 0.2] | <0.001 | 0.249 [0.208; 0.290] |
| Drop MAP | 43.5 [33.7; 54.4] | 42.0 [32.4; 53.0] | 46.5 [36.5; 56.8] | <0.001 | 0.241 [0.200; 0.284] |
| Max SAP | 145.9 [133.7; 157.4] | 144.4 [132.2; 156.4] | 148.7 [136.9; 159.0] | <0.001 | 0.217 [0.179; 0.257] |
| Var MAP | 0.1 [0.1; 0.2] | 0.1 [0.1; 0.1] | 0.1 [0.1; 0.2] | <0.001 | 0.188 [0.143; 0.230] |
| Std MAP | 10.0 [7.7; 12.7] | 9.7 [7.5; 12.5] | 10.5 [8.1; 13.2] | <0.001 | 0.185 [0.140; 0.229] |
| Max MAP | 108.0 [99.0; 117.7] | 107.4 [98.5; 117.1] | 109.4 [99.9; 118.8] | <0.001 | 0.132 [0.090; 0.172] |
| Drop DAP | 30.1 [24.7; 35.3] | 29.9 [24.4; 34.9] | 30.6 [25.3; 35.9] | <0.001 | 0.100 [0.057; 0.143] |
| Var DAP | 0.1 [0.1; 0.1] | 0.1 [0.1; 0.1] | 0.1 [0.1; 0.1] | 0.373 | 0.022 [0.001; 0.065] |
| Std DAP | 7.1 [5.6; 8.8] | 7.1 [5.6; 8.8] | 7.1 [5.5; 8.8] | 0.856 | 0.005 [0.001; 0.050] |

Results are presented as median and interquartile range (IQR),

aSMD: Absolute standardized mean distance per cluster

aSMD reported were computed over the entire dataset and their CI computed from a 2000 iteration bootstrap. Note that all variables including areas under the curve were removed as statistically redundant with cumulative time variables.

Drop PP: maximum – minimum PP computed over the entire intervention

std PP: standard deviation of pulse pressure

Drop SAP: maximum – minimum PP computed over the entire intervention

Var PP: variability of pulse pressure

Max PP: maximal value of pulse pressure

std SAP: standard deviation of systolic arterial pressure

Var SAP: variability of systolic arterial pressure

Drop MAP: maximum – minimum MAP computed over the entire intervention.

Max SAP: maximal value of maximal value of pulse pressure

Var MAP: variability of mean arterial pressure

std MAP: standard deviation of mean arterial pressure

Max MAP: maximal value of mean arterial pressure

Drop DAP: maximum – minimum DAP computed over the entire intervention.

Var DAP: variability of diastolic arterial pressure

std DAP: standard deviation of diastolic arterial pressure

**Absolute standardized mean distance per cluster for Cluster 2**

| Cluster 2 |  |  |  |  |  |
| --- | --- | --- | --- | --- | --- |
| Variables | **Overall (9,516)** | **No pLOS (6,289)** | **pLOS (3,227)** | **p-val** | **aSMD[95%CI]** |
| CumTimePP>61 | 56.4 [46.7; 60.1] | 57.1 [48.5; 60.2] | 54.5 [43.1; 59.7] | <0.001 | 0.209 [0.165; 0.252] |
| CumTimePP>60 | 55.6 [45.3; 60.1] | 56.5 [47.2; 60.2] | 53.7 [41.6; 59.4] | <0.001 | 0.206 [0.163; 0.251] |
| CumTimePP>59 | 54.9 [43.7; 60.0] | 55.7 [45.7; 60.1] | 52.8 [40.1; 59.0] | <0.001 | 0.204 [0.160; 0.248] |
| CumTimePP>58 | 54.1 [41.8; 59.5] | 55.1 [43.9; 60.0] | 51.7 [38.3; 58.5] | <0.001 | 0.200 [0.157; 0.243] |
| CumTimePP>57 | 53.2 [40.0; 59.1] | 54.2 [42.2; 59.6] | 50.4 [36.4; 57.9] | <0.001 | 0.198 [0.155; 0.241] |
| CumTimePP>56 | 52.2 [38.0; 58.6] | 53.2 [40.2; 59.1] | 49.3 [34.6; 57.3] | <0.001 | 0.194 [0.151; 0.236] |
| CumTimePP>55 | 50.9 [35.8; 58.0] | 52.1 [38.0; 58.6] | 47.8 [32.7; 56.7] | <0.001 | 0.187 [0.145; 0.231] |
| CumTimePP>54 | 49.5 [33.6; 57.4] | 50.8 [35.6; 58.0] | 46.4 [30.6; 56.1] | <0.001 | 0.180 [0.138; 0.224] |
| CumTimePP>53 | 48.1 [31.2; 56.7] | 49.3 [32.9; 57.3] | 44.9 [28.3; 55.3] | <0.001 | 0.173 [0.130; 0.217] |
| Min PP | 32.6 [28.4; 37.3] | 33.0 [28.8; 37.7] | 31.7 [27.4; 36.3] | <0.001 | 0.170 [0.128; 0.212] |
| CumTimePP>52 | 46.4 [28.8; 55.9] | 47.8 [30.5; 56.6] | 43.1 [26.1; 54.5] | <0.001 | 0.165 [0.121; 0.208] |
| CumTimePP>51 | 44.5 [26.2; 54.9] | 45.9 [27.8; 55.6] | 41.2 [24.0; 53.3] | <0.001 | 0.156 [0.113; 0.199] |
| CumTimePP>50 | 42.4 [23.7; 53.8] | 43.9 [25.1; 54.5] | 39.4 [21.6; 52.1] | <0.001 | 0.147 [0.104; 0.190] |
| Mean PP | 46.9 [42.1; 53.6] | 46.5 [41.8; 52.9] | 47.9 [42.6; 54.7] | <0.001 | 0.147 [0.103; 0.190] |
| CumTimePP>49 | 40.1 [21.3; 52.5] | 41.5 [22.4; 53.4] | 37.2 [19.5; 50.5] | <0.001 | 0.139 [0.096; 0.180] |
| Median PP | 45.6 [40.8; 52.6] | 45.2 [40.6; 51.9] | 46.4 [41.3; 53.8] | <0.001 | 0.133 [0.089; 0.175] |
| CumTimePP>48 | 37.4 [18.8; 51.1] | 39.0 [19.8; 51.9] | 34.7 [17.6; 48.9] | <0.001 | 0.127 [0.084; 0.168] |
| CumTimePP>47 | 34.7 [16.6; 49.3] | 36.1 [17.2; 50.3] | 31.9 [15.5; 47.1] | <0.001 | 0.118 [0.077; 0.159] |
| CumTimePP>46 | 31.5 [14.5; 47.3] | 32.9 [14.9; 48.3] | 29.1 [13.5; 44.8] | <0.001 | 0.110 [0.069; 0.150] |
| CumTimePP>29 | 0.0 [0.0; 0.5] | 0.0 [0.0; 0.2] | 0.0 [0.0; 0.9] | <0.001 | 0.110 [0.065; 0.153] |
| CumTimePP>30 | 0.0 [0.0; 1.1] | 0.0 [0.0; 0.9] | 0.0 [0.0; 1.7] | <0.001 | 0.109 [0.064; 0.150] |
| CumTimePP>45 | 28.2 [12.0; 44.8] | 29.3 [12.3; 46.0] | 26.7 [11.5; 42.5] | <0.001 | 0.098 [0.056; 0.138] |
| CumTimePP>31 | 0.0 [0.0; 2.1] | 0.0 [0.0; 1.7] | 0.0 [0.0; 2.7] | <0.001 | 0.096 [0.053; 0.136] |
| CumTimePP>32 | 0.0 [0.0; 3.3] | 0.0 [0.0; 2.9] | 0.3 [0.0; 4.1] | <0.001 | 0.091 [0.048; 0.133] |
| CumTimePP>44 | 25.0 [9.9; 42.0] | 25.6 [10.0; 43.2] | 23.5 [9.8; 39.4] | <0.001 | 0.084 [0.043; 0.124] |
| CumTimePP>33 | 0.4 [0.0; 4.9] | 0.0 [0.0; 4.6] | 0.9 [0.0; 5.8] | <0.001 | 0.078 [0.034; 0.119] |
| CumTimePP>43 | 21.3 [7.9; 38.7] | 21.8 [7.8; 39.9] | 20.4 [7.9; 36.5] | 0.011 | 0.068 [0.027; 0.108] |
| CumTimePP>34 | 1.2 [0.0; 6.8] | 0.9 [0.0; 6.4] | 1.6 [0.0; 7.7] | <0.001 | 0.062 [0.020; 0.103] |
| CumTimePP>42 | 18.2 [6.0; 35.0] | 18.3 [5.8; 36.1] | 18.0 [6.3; 32.6] | 0.133 | 0.052 [0.013; 0.093] |
| CumTimePP>35 | 2.2 [0.0; 9.3] | 1.9 [0.0; 8.9] | 2.8 [0.0; 9.9] | <0.001 | 0.046 [0.005; 0.088] |
| CumTimePP>41 | 15.0 [4.2; 31.0] | 14.9 [4.0; 32.0] | 15.0 [4.9; 28.7] | 0.675 | 0.037 [0.004; 0.079] |
| CumTimePP>36 | 3.5 [0.0; 12.2] | 3.2 [0.0; 12.0] | 4.1 [0.0; 12.7] | <0.001 | 0.031 [0.002; 0.074] |
| CumTimePP>40 | 12.1 [2.9; 26.9] | 11.9 [2.6; 27.8] | 12.4 [3.6; 25.3] | 0.566 | 0.022 [0.001; 0.064] |
| CumTimePP>37 | 5.3 [0.0; 15.6] | 4.9 [0.0; 15.5] | 5.9 [0.6; 15.8] | 0.001 | 0.019 [0.001; 0.061] |
| CumTimePP>38 | 7.3 [0.7; 19.1] | 7.1 [0.4; 19.3] | 8.0 [1.4; 18.8] | 0.010 | 0.008 [0.001; 0.051] |
| CumTimePP>39 | 9.6 [1.7; 23.1] | 9.3 [1.3; 23.5] | 10.2 [2.4; 22.0] | 0.122 | 0.007 [0.001; 0.052] |

aSMD: Absolute standardized mean distance per cluster

aSMD reported were computed over the entire dataset and their CI computed from a 2000 iteration bootstrap. Note that all variables including areas under the curve were removed as statistically redundant with cumulative time variables.

CumTimePP>XX: cumulative time of pulse pressure under XX mmHg

Min PP: minimal value of pulse pressure

Mean PP: mean value of all pulse pressure measurements during anesthesia

Median PP: median value of all pulse pressure measurements during anesthesia

**Absolute standardized mean distance per cluster for Cluster 3**

| Cluster 3 |  |  |  |  |  |
| --- | --- | --- | --- | --- | --- |
| Variables | **Overall (9,516)** | **No pLOS (6,289)** | **pLOS(3,227)** | **p-val** | aSMD [95%CI] |
| Min MAP | 63.5 [58.2; 69.7] | 64.0 [58.9; 70.3] | 62.4 [57.0; 68.1] | <0.001 | 0.200 [0.160; 0.242] |
| Min SAP | 86.2 [80.8; 94.6] | 87.0 [81.2; 95.6] | 84.9 [80.0; 92.6] | <0.001 | 0.183 [0.144; 0.223] |
| Min DAP | 50.9 [47.4; 54.2] | 51.0 [47.7; 54.5] | 50.5 [46.9; 53.7] | <0.001 | 0.135 [0.095; 0.178] |
| CumTimeMAP>59 | 0.0 [0.0; 0.5] | 0.0 [0.0; 0.2] | 0.0 [0.0; 0.9] | <0.001 | 0.102 [0.059; 0.148] |
| CumTimeMAP>60 | 0.0 [0.0; 1.0] | 0.0 [0.0; 0.8] | 0.0 [0.0; 1.5] | <0.001 | 0.098 [0.056; 0.143] |
| CumTimeMAP>61 | 0.0 [0.0; 1.7] | 0.0 [0.0; 1.4] | 0.0 [0.0; 2.1] | <0.001 | 0.090 [0.048; 0.134] |
| CumTimeMAP>62 | 0.0 [0.0; 2.5] | 0.0 [0.0; 2.2] | 0.0 [0.0; 3.0] | <0.001 | 0.076 [0.033; 0.118] |
| CumTimeMAP>63 | 0.0 [0.0; 3.5] | 0.0 [0.0; 3.2] | 0.4 [0.0; 4.0] | <0.001 | 0.068 [0.027; 0.111] |
| CumTimeSAP>82 | 0.0 [0.0; 0.8] | 0.0 [0.0; 0.6] | 0.0 [0.0; 1.1] | <0.001 | 0.053 [0.010; 0.097] |
| CumTimeMAP>64 | 0.5 [0.0; 4.9] | 0.0 [0.0; 4.5] | 1.1 [0.0; 5.4] | <0.001 | 0.049 [0.009; 0.091] |
| CumTimeSAP>83 | 0.0 [0.0; 1.3] | 0.0 [0.0; 1.1] | 0.0 [0.0; 1.7] | <0.001 | 0.048 [0.009; 0.091] |
| CumTimeSAP>84 | 0.0 [0.0; 2.0] | 0.0 [0.0; 1.8] | 0.0 [0.0; 2.4] | <0.001 | 0.046 [0.008; 0.087] |
| CumTimeSAP>85 | 0.0 [0.0; 2.8] | 0.0 [0.0; 2.6] | 0.1 [0.0; 3.2] | <0.001 | 0.040 [0.005; 0.081] |
| CumTimeSAP>86 | 0.0 [0.0; 3.8] | 0.0 [0.0; 3.6] | 0.6 [0.0; 4.2] | <0.001 | 0.036 [0.003; 0.078] |
| CumTimeDAP>50 | 0.0 [0.0; 2.3] | 0.0 [0.0; 2.1] | 0.0 [0.0; 2.8] | <0.001 | 0.036 [0.003; 0.075] |
| CumTimeMAP>65 | 1.3 [0.0; 6.5] | 0.9 [0.0; 6.2] | 1.9 [0.0; 7.0] | <0.001 | 0.034 [0.003; 0.077] |
| CumTimeSAP>87 | 0.5 [0.0; 4.9] | 0.0 [0.0; 4.7] | 1.1 [0.0; 5.3] | <0.001 | 0.029 [0.002; 0.072] |
| CumTimeDAP>49 | 0.0 [0.0; 1.3] | 0.0 [0.0; 1.1] | 0.0 [0.0; 1.7] | <0.001 | 0.029 [0.001; 0.068] |
| CumTimeMAP>66 | 2.2 [0.0; 8.4] | 1.8 [0.0; 8.1] | 2.8 [0.0; 9.0] | <0.001 | 0.024 [0.001; 0.069] |
| CumTimeSAP>88 | 1.1 [0.0; 6.2] | 0.7 [0.0; 6.0] | 1.6 [0.0; 6.5] | <0.001 | 0.023 [0.001; 0.066] |
| CumTimeSAP>89 | 1.7 [0.0; 7.5] | 1.4 [0.0; 7.3] | 2.2 [0.0; 7.9] | <0.001 | 0.022 [0.001; 0.065] |
| CumTimeSAP>90 | 2.5 [0.0; 8.9] | 2.1 [0.0; 8.7] | 3.1 [0.0; 9.3] | <0.001 | 0.021 [0.001; 0.064] |
| CumTimeDAP>58 | 17.0 [6.0; 33.5] | 16.7 [5.7; 33.6] | 17.6 [6.6; 33.0] | 0.092 | 0.020 [0.001; 0.062] |
| CumTimeMAP>67 | 3.3 [0.0; 10.6] | 2.9 [0.0; 10.4] | 4.1 [0.0; 11.0] | <0.001 | 0.018 [0.001; 0.061] |
| CumTimeMAP>68 | 4.6 [0.0; 12.9] | 4.2 [0.0; 12.8] | 5.3 [0.0; 13.0] | <0.001 | 0.014 [0.001; 0.055] |
| CumTimeMAP>69 | 5.9 [0.0; 15.1] | 5.5 [0.0; 15.0] | 6.7 [0.9; 15.3] | <0.001 | 0.014 [0.001; 0.055] |
| CumTimeDAP>57 | 13.8 [4.2; 28.8] | 13.4 [4.0; 29.2] | 14.4 [4.8; 28.6] | 0.096 | 0.013 [0.001; 0.056] |
| CumTimeSAP>91 | 3.3 [0.0; 10.5] | 2.9 [0.0; 10.3] | 3.9 [0.0; 10.9] | <0.001 | 0.013 [0.001; 0.056] |
| CumTimeSAP>92 | 4.2 [0.0; 12.3] | 3.8 [0.0; 12.2] | 4.8 [0.0; 12.4] | <0.001 | 0.013 [0.001; 0.055] |
| CumTimeMAP>70 | 7.4 [0.4; 17.8] | 6.9 [0.0; 17.8] | 8.1 [1.6; 17.8] | <0.001 | 0.013 [0.001; 0.054] |
| CumTimeSAP>93 | 5.3 [0.0; 14.0] | 5.0 [0.0; 14.0] | 5.9 [0.4; 14.0] | <0.001 | 0.012 [0.001; 0.054] |
| CumTimeDAP>51 | 0.3 [0.0; 4.6] | 0.0 [0.0; 4.5] | 0.6 [0.0; 4.8] | <0.001 | 0.012 [0.001; 0.051] |
| CumTimeMAP>71 | 8.8 [1.5; 20.4] | 8.3 [0.8; 20.5] | 9.6 [2.4; 20.3] | 0.001 | 0.010 [0.001; 0.052] |
| CumTimeSAP>94 | 6.4 [0.0; 15.9] | 6.0 [0.0; 16.0] | 7.0 [0.9; 15.8] | <0.001 | 0.009 [0.001; 0.050] |
| CumTimeDAP>53 | 3.7 [0.0; 11.7] | 3.5 [0.0; 11.9] | 4.0 [0.0; 11.5] | 0.018 | 0.007 [0.001; 0.051] |
| CumTimeMAP>72 | 10.4 [2.4; 22.9] | 10.0 [1.9; 22.9] | 11.2 [3.3; 22.8] | 0.005 | 0.007 [0.001; 0.050] |
| CumTimeSAP>98 | 11.0 [2.7; 23.7] | 10.6 [2.2; 24.3] | 11.5 [3.6; 23.2] | 0.027 | 0.007 [0.001; 0.049] |
| CumTimeDAP>54 | 5.8 [0.0; 15.9] | 5.7 [0.0; 16.0] | 6.1 [0.5; 15.9] | 0.043 | 0.006 [0.001; 0.049] |
| CumTimeDAP>56 | 10.8 [2.6; 24.3] | 10.5 [2.2; 24.5] | 11.3 [3.1; 24.0] | 0.108 | 0.005 [0.001; 0.050] |
| CumTimeSAP>95 | 7.4 [0.4; 17.8] | 7.0 [0.0; 17.9] | 8.0 [1.5; 17.4] | <0.001 | 0.005 [0.001; 0.050] |
| CumTimeMAP>73 | 12.2 [3.6; 25.6] | 11.8 [3.0; 25.8] | 12.7 [4.5; 25.2] | 0.023 | 0.004 [0.001; 0.048] |
| CumTimeSAP>97 | 9.7 [1.9; 21.6] | 9.4 [1.4; 22.1] | 10.3 [2.9; 21.0] | 0.009 | 0.004 [0.001; 0.048] |
| CumTimeDAP>52 | 1.8 [0.0; 7.8] | 1.7 [0.0; 7.7] | 2.1 [0.0; 7.8] | 0.010 | 0.003 [0.001; 0.047] |
| CumTimeDAP>55 | 8.1 [1.1; 20.0] | 7.8 [0.8; 20.0] | 8.6 [1.5; 20.0] | 0.054 | 0.002 [0.001; 0.048] |
| CumTimeSAP>96 | 8.5 [1.1; 19.7] | 8.1 [0.5; 20.0] | 9.2 [2.1; 19.0] | 0.001 | 0.001 [0.001; 0.046] |
| CumTimeMAP>74 | 14.0 [4.8; 28.4] | 13.6 [4.2; 28.8] | 14.6 [5.7; 27.8] | 0.086 | 0.001 [0.001; 0.047] |

aSMD: Absolute standardized mean distance per cluster

aSMD reported were computed over the entire dataset and their CI computed from a 2000 iteration bootstrap. Note that all variables including areas under the curve were removed as statistically redundant with cumulative time variables.

Min MAP: minimal value of mean arterial pressure

Min SAP: minimal value of systolic arterial pressure

Min DAP: minimal value of diastolic arterial pressure

CumTime X>Y: Cumulative time the variable X (systolic, diastolic, mean, or pulse pressure) spent below a value Y.

**Absolute standardized mean distance per cluster for Cluster 4**

| Cluster 4 |  |  |  |  |  |
| --- | --- | --- | --- | --- | --- |
| Variables | **Overall (9,516)** | **No pLOS (6,289)** | **pLOS(3,227)** | **p-val** | **aSMD[95%CI]** |
| CumTimeSAP>131 | 54.3 [44.9; 59.2] | 54.9 [45.8; 59.7] | 53.1 [43.1; 58.2] | <0.001 | 0.121 [0.078; 0.164] |
| CumTimeSAP>132 | 54.8 [45.9; 59.5] | 55.3 [46.8; 60.0] | 53.8 [44.1; 58.6] | <0.001 | 0.121 [0.080; 0.165] |
| CumTimeSAP>130 | 53.6 [43.9; 58.9] | 54.3 [44.8; 59.3] | 52.5 [42.1; 57.9] | <0.001 | 0.118 [0.076; 0.162] |
| CumTimeSAP>129 | 53.0 [42.7; 58.5] | 53.6 [43.6; 59.0] | 51.9 [41.2; 57.7] | <0.001 | 0.115 [0.072; 0.158] |
| CumTimeSAP>128 | 52.3 [41.5; 58.1] | 52.9 [42.5; 58.6] | 51.1 [40.0; 57.2] | <0.001 | 0.112 [0.069; 0.154] |
| CumTimeSAP>127 | 51.6 [40.2; 57.8] | 52.2 [41.1; 58.2] | 50.4 [38.7; 56.8] | <0.001 | 0.108 [0.066; 0.150] |
| CumTimeSAP>126 | 51.0 [39.0; 57.4] | 51.5 [39.6; 57.8] | 49.5 [37.5; 56.4] | <0.001 | 0.106 [0.064; 0.149] |
| CumTimeSAP>125 | 50.2 [37.7; 57.0] | 50.7 [38.3; 57.5] | 48.8 [36.2; 55.9] | <0.001 | 0.104 [0.061; 0.147] |
| CumTimeSAP>124 | 49.4 [36.3; 56.5] | 50.0 [36.9; 57.0] | 47.9 [34.8; 55.4] | <0.001 | 0.100 [0.058; 0.142] |
| CumTimeSAP>123 | 48.4 [34.6; 56.0] | 49.1 [35.4; 56.5] | 46.9 [33.5; 54.9] | <0.001 | 0.095 [0.053; 0.137] |
| CumTimeSAP>122 | 47.5 [33.1; 55.5] | 48.2 [33.8; 56.0] | 46.0 [32.1; 54.3] | <0.001 | 0.092 [0.051; 0.135] |
| CumTimeSAP>121 | 46.4 [31.7; 54.9] | 47.1 [32.2; 55.4] | 45.0 [30.7; 53.7] | <0.001 | 0.089 [0.048; 0.131] |
| CumTimeSAP>120 | 45.3 [30.2; 54.3] | 46.1 [30.6; 54.9] | 43.8 [29.3; 53.1] | <0.001 | 0.085 [0.044; 0.126] |
| CumTimeSAP>119 | 44.2 [28.6; 53.7] | 44.9 [28.9; 54.2] | 42.8 [27.8; 52.3] | <0.001 | 0.079 [0.038; 0.119] |
| CumTimeSAP>118 | 42.8 [27.1; 52.9] | 43.7 [27.2; 53.5] | 41.7 [26.6; 51.6] | <0.001 | 0.074 [0.033; 0.114] |
| CumTimeSAP>117 | 41.6 [25.5; 52.2] | 42.3 [25.6; 52.8] | 40.5 [25.4; 50.8] | <0.001 | 0.069 [0.029; 0.110] |
| CumTimeSAP>116 | 40.2 [24.0; 51.3] | 40.9 [24.0; 52.0] | 38.9 [23.8; 49.9] | <0.001 | 0.066 [0.023; 0.105] |
| CumTimeDAP>68 | 44.8 [27.5; 54.3] | 44.3 [26.8; 54.2] | 45.7 [29.0; 54.6] | 0.009 | 0.064 [0.024; 0.106] |
| CumTimeDAP>67 | 42.5 [25.1; 53.2] | 42.0 [24.5; 53.1] | 43.5 [26.3; 53.5] | 0.010 | 0.063 [0.023; 0.105] |
| CumTimeDAP>66 | 40.3 [22.5; 52.0] | 39.7 [21.9; 51.8] | 41.2 [23.9; 52.3] | 0.011 | 0.061 [0.022; 0.104] |
| CumTimeDAP>65 | 37.7 [20.2; 50.6] | 37.0 [19.8; 50.5] | 38.8 [21.1; 50.9] | 0.011 | 0.061 [0.021; 0.102] |
| CumTimeDAP>69 | 46.7 [30.2; 55.3] | 46.4 [29.6; 55.1] | 47.3 [31.3; 55.6] | 0.013 | 0.061 [0.019; 0.104] |
| Mean SAP | 111.2 [103.6; 119.8] | 111.0 [103.1; 119.5] | 111.5 [104.2; 120.1] | 0.003 | 0.061 [0.018; 0.102] |
| CumTimeSAP>115 | 38.8 [22.4; 50.6] | 39.4 [22.4; 51.2] | 37.6 [22.3; 49.1] | <0.001 | 0.061 [0.018; 0.101] |
| CumTimeDAP>70 | 48.5 [32.8; 56.2] | 48.2 [32.2; 56.1] | 49.1 [34.3; 56.5] | 0.017 | 0.060 [0.018; 0.102] |
| CumTimeDAP>71 | 50.0 [35.5; 57.0] | 49.8 [35.0; 56.9] | 50.6 [36.7; 57.2] | 0.023 | 0.059 [0.017; 0.102] |
| CumTimeDAP>72 | 51.4 [38.0; 57.8] | 51.1 [37.4; 57.7] | 51.9 [39.0; 57.9] | 0.027 | 0.059 [0.017; 0.102] |
| CumTimeSAP>114 | 37.2 [20.7; 49.6] | 38.0 [20.7; 50.3] | 35.9 [20.9; 48.2] | <0.001 | 0.059 [0.016; 0.098] |
| CumTimeDAP>64 | 34.9 [18.0; 49.0] | 34.3 [17.7; 48.8] | 36.3 [19.0; 49.3] | 0.014 | 0.058 [0.017; 0.100] |
| CumTimeDAP>73 | 52.7 [40.3; 58.5] | 52.4 [39.9; 58.3] | 53.1 [41.1; 58.5] | 0.034 | 0.056 [0.016; 0.099] |
| CumTimeSAP>113 | 35.6 [19.3; 48.6] | 36.2 [19.2; 49.3] | 34.6 [19.6; 47.2] | 0.002 | 0.055 [0.012; 0.093] |
| Mean DAP | 63.3 [58.8; 68.7] | 63.5 [58.7; 68.9] | 63.0 [58.8; 68.2] | 0.024 | 0.054 [0.014; 0.096] |
| CumTimeDAP>63 | 32.1 [15.9; 47.2] | 31.4 [15.4; 47.1] | 33.4 [16.7; 47.4] | 0.019 | 0.054 [0.013; 0.095] |
| CumTimeDAP>74 | 53.8 [42.6; 59.0] | 53.6 [42.2; 59.0] | 54.0 [43.2; 59.1] | 0.078 | 0.052 [0.012; 0.093] |
| Median DAP | 62.3 [57.3; 68.9] | 62.5 [57.2; 69.2] | 61.8 [57.3; 68.3] | 0.043 | 0.051 [0.012; 0.094] |
| CumTimeDAP>62 | 29.1 [13.8; 45.2] | 28.4 [13.4; 45.2] | 30.5 [14.7; 45.3] | 0.020 | 0.051 [0.011; 0.092] |
| CumTimeSAP>112 | 33.9 [17.9; 47.4] | 34.6 [17.8; 48.2] | 33.0 [18.1; 45.9] | 0.005 | 0.051 [0.009; 0.090] |
| CumTimeDAP>75 | 54.9 [44.7; 59.5] | 54.7 [44.3; 59.4] | 55.1 [45.4; 59.6] | 0.158 | 0.050 [0.011; 0.091] |
| Median SAP | 109.7 [100.9; 119.7] | 109.5 [100.6; 119.6] | 110.0 [101.4; 120.1] | 0.025 | 0.050 [0.006; 0.093] |
| CumTimeSAP>111 | 32.2 [16.6; 46.2] | 32.9 [16.5; 47.0] | 31.4 [16.9; 44.9] | 0.012 | 0.048 [0.008; 0.087] |
| CumTimeDAP>61 | 26.1 [11.7; 42.9] | 25.5 [11.3; 42.8] | 27.4 [12.6; 42.9] | 0.029 | 0.045 [0.006; 0.087] |
| CumTimeSAP>110 | 30.5 [15.3; 45.0] | 30.9 [15.1; 45.7] | 29.6 [15.7; 43.6] | 0.030 | 0.044 [0.006; 0.083] |
| CumTimeSAP>109 | 28.6 [14.0; 43.7] | 28.8 [13.7; 44.4] | 28.1 [14.3; 42.3] | 0.067 | 0.040 [0.003; 0.080] |
| CumTimeSAP>107 | 25.0 [11.7; 40.8] | 25.2 [11.4; 41.7] | 24.8 [12.2; 39.1] | 0.161 | 0.039 [0.002; 0.079] |
| CumTimeSAP>106 | 23.2 [10.5; 39.3] | 23.2 [10.3; 40.1] | 23.0 [11.1; 37.6] | 0.231 | 0.038 [0.002; 0.078] |
| CumTimeSAP>108 | 26.9 [12.9; 42.3] | 27.0 [12.6; 43.1] | 26.6 [13.4; 40.8] | 0.125 | 0.038 [0.002; 0.078] |
| CumTimeDAP>60 | 23.1 [9.8; 40.4] | 22.6 [9.4; 40.5] | 24.4 [10.4; 40.3] | 0.044 | 0.037 [0.003; 0.078] |
| CumTimeSAP>105 | 21.4 [9.4; 37.5] | 21.4 [9.1; 38.4] | 21.4 [9.9; 36.2] | 0.348 | 0.037 [0.002; 0.078] |
| CumTimeSAP>104 | 19.8 [8.3; 35.8] | 19.8 [8.0; 36.6] | 19.8 [9.0; 34.4] | 0.635 | 0.032 [0.002; 0.073] |
| CumTimeDAP>59 | 20.1 [7.8; 37.2] | 19.7 [7.6; 37.4] | 20.9 [8.5; 36.9] | 0.067 | 0.028 [0.001; 0.070] |
| CumTimeSAP>103 | 18.2 [7.3; 34.0] | 18.2 [6.8; 34.7] | 18.4 [8.1; 32.6] | 0.991 | 0.026 [0.002; 0.068] |
| CumTimeSAP>102 | 16.7 [6.3; 32.1] | 16.6 [5.9; 32.9] | 17.0 [7.0; 31.1] | 0.678 | 0.022 [0.001; 0.064] |
| CumTimeMAP>96 | 53.7 [44.2; 58.8] | 53.9 [44.5; 59.1] | 53.3 [44.1; 58.3] | 0.001 | 0.021 [0.001; 0.062] |
| CumTimeMAP>98 | 55.3 [47.3; 59.7] | 55.5 [47.5; 60.0] | 54.8 [47.0; 59.3] | <0.001 | 0.021 [0.001; 0.061] |
| CumTimeMAP>97 | 54.5 [45.9; 59.3] | 54.7 [46.2; 59.5] | 54.1 [45.6; 58.8] | <0.001 | 0.021 [0.001; 0.060] |
| CumTimeMAP>95 | 52.9 [42.7; 58.3] | 53.0 [42.7; 58.6] | 52.5 [42.6; 57.9] | 0.001 | 0.020 [0.001; 0.060] |
| CumTimeMAP>94 | 51.9 [40.8; 57.8] | 52.1 [40.8; 58.0] | 51.5 [40.7; 57.3] | 0.004 | 0.019 [0.001; 0.060] |
| CumTimeSAP>101 | 15.2 [5.3; 30.0] | 15.0 [4.9; 30.7] | 15.6 [6.1; 29.3] | 0.394 | 0.018 [0.001; 0.060] |
| CumTimeMAP>93 | 50.9 [38.9; 57.3] | 51.1 [38.9; 57.5] | 50.6 [38.9; 56.8] | 0.010 | 0.016 [0.001; 0.056] |
| CumTimeSAP>100 | 13.7 [4.4; 28.1] | 13.4 [4.0; 28.5] | 14.3 [5.3; 27.1] | 0.206 | 0.014 [0.001; 0.055] |
| CumTimeMAP>92 | 49.8 [37.1; 56.7] | 50.0 [36.9; 56.9] | 49.4 [37.4; 56.2] | 0.022 | 0.013 [0.001; 0.054] |
| CumTimeMAP>80 | 28.1 [13.7; 43.2] | 28.1 [13.2; 43.8] | 28.0 [14.4; 42.2] | 0.625 | 0.013 [0.001; 0.051] |
| CumTimeMAP>79 | 25.5 [12.1; 41.3] | 25.5 [11.7; 41.9] | 25.6 [12.8; 40.1] | 0.828 | 0.012 [0.001; 0.051] |
| CumTimeMAP>88 | 44.6 [29.0; 53.7] | 44.9 [28.8; 54.0] | 44.2 [29.6; 53.1] | 0.093 | 0.012 [0.001; 0.050] |
| CumTimeMAP>91 | 48.7 [35.2; 56.0] | 48.9 [35.1; 56.2] | 48.4 [35.5; 55.6] | 0.046 | 0.011 [0.001; 0.052] |
| CumTimeSAP>99 | 12.3 [3.6; 26.0] | 11.9 [3.1; 26.4] | 12.8 [4.6; 25.1] | 0.092 | 0.011 [0.001; 0.052] |
| CumTimeMAP>87 | 43.0 [27.0; 52.7] | 43.2 [26.7; 53.1] | 42.3 [27.7; 52.1] | 0.132 | 0.011 [0.001; 0.051] |
| CumTimeMAP>81 | 30.5 [15.4; 45.0] | 30.6 [15.1; 45.5] | 30.2 [16.1; 43.7] | 0.508 | 0.011 [0.001; 0.049] |
| CumTimeMAP>83 | 35.1 [19.0; 48.0] | 35.1 [18.8; 48.5] | 35.2 [19.9; 47.1] | 0.327 | 0.011 [0.001; 0.049] |
| Mean MAP | 82.0 [76.4; 88.7] | 82.0 [76.3; 88.7] | 82.0 [76.7; 88.4] | 0.514 | 0.010 [0.001; 0.051] |
| CumTimeMAP>86 | 41.0 [24.8; 51.7] | 41.4 [24.5; 52.1] | 40.6 [25.7; 51.1] | 0.180 | 0.010 [0.001; 0.049] |
| CumTimeMAP>89 | 46.1 [31.2; 54.5] | 46.3 [31.0; 54.7] | 45.7 [31.6; 54.0] | 0.086 | 0.010 [0.001; 0.049] |
| CumTimeMAP>82 | 32.9 [17.1; 46.6] | 32.9 [16.7; 47.1] | 32.8 [18.0; 45.5] | 0.437 | 0.010 [0.001; 0.048] |
| CumTimeMAP>85 | 39.1 [22.8; 50.7] | 39.2 [22.6; 51.1] | 38.8 [23.7; 50.0] | 0.222 | 0.010 [0.001; 0.048] |
| CumTimeMAP>78 | 23.1 [10.5; 39.0] | 23.1 [10.0; 39.7] | 23.1 [11.4; 37.9] | 0.890 | 0.009 [0.001; 0.049] |
| CumTimeMAP>90 | 47.4 [33.2; 55.3] | 47.7 [33.1; 55.5] | 47.0 [33.7; 54.9] | 0.065 | 0.009 [0.001; 0.049] |
| CumTimeMAP>84 | 37.2 [20.9; 49.5] | 37.2 [20.5; 49.9] | 37.1 [21.8; 48.6] | 0.286 | 0.009 [0.001; 0.047] |
| Median MAP | 80.8 [74.6; 88.5] | 80.8 [74.5; 88.6] | 80.9 [74.9; 88.2] | 0.595 | 0.008 [0.001; 0.050] |
| CumTimeMAP>77 | 20.8 [8.9; 36.4] | 20.7 [8.5; 37.1] | 20.8 [9.8; 35.5] | 0.660 | 0.008 [0.001; 0.048] |
| CumTimeMAP>76 | 18.5 [7.5; 33.8] | 18.3 [7.0; 34.3] | 18.7 [8.2; 32.9] | 0.442 | 0.006 [0.000; 0.047] |
| CumTimeMAP>75 | 16.2 [6.1; 31.2] | 15.9 [5.6; 31.5] | 16.7 [6.9; 30.4] | 0.224 | 0.003 [0.001; 0.045] |
| Max DAP | 82.3 [76.2; 87.3] | 82.3 [76.3; 87.2] | 82.2 [76.1; 87.4] | 0.912 | 0.001 [0.001; 0.048] |

aSMD: Absolute standardized mean distance per cluster

aSMD reported were computed over the entire dataset and their CI computed from a 2000 iteration bootstrap. Note that all variables including areas under the curve were removed as statistically redundant with cumulative time variables.

CumTimeX>Y: Cumulative time the variable X (systolic, diastolic, mean, or pulse pressure) spent below a value Y.

Mean SAP: mean value of all systolic pressure measurements

Mean DAP: mean value of all diastolic pressure measurements

Median DAP: median value of all diastolic pressure measurements

Median SAP: median value of all diastolic pressure measurements

Mean MAP: mean value of all mean pressure measurements

Median MAP: median value of all mean pressure measurements

Max DAP: maximal value of diastolic pressure measurements*.*
